# Supplementary figures and images for: Outbreak of cutaneous leishmaniasis amongst militia members in a non-endemic district under conflict in the lowlands of Somali Region caused by Leishmania tropica, Eastern Ethiopia
Source: PLoS Negl Trop Dis. 2025 Jul 22;19(7):e0013246. doi: 10.1371/journal.pntd.0013246 (PMC12324667; doi:10.1371/journal.pntd.0013246)

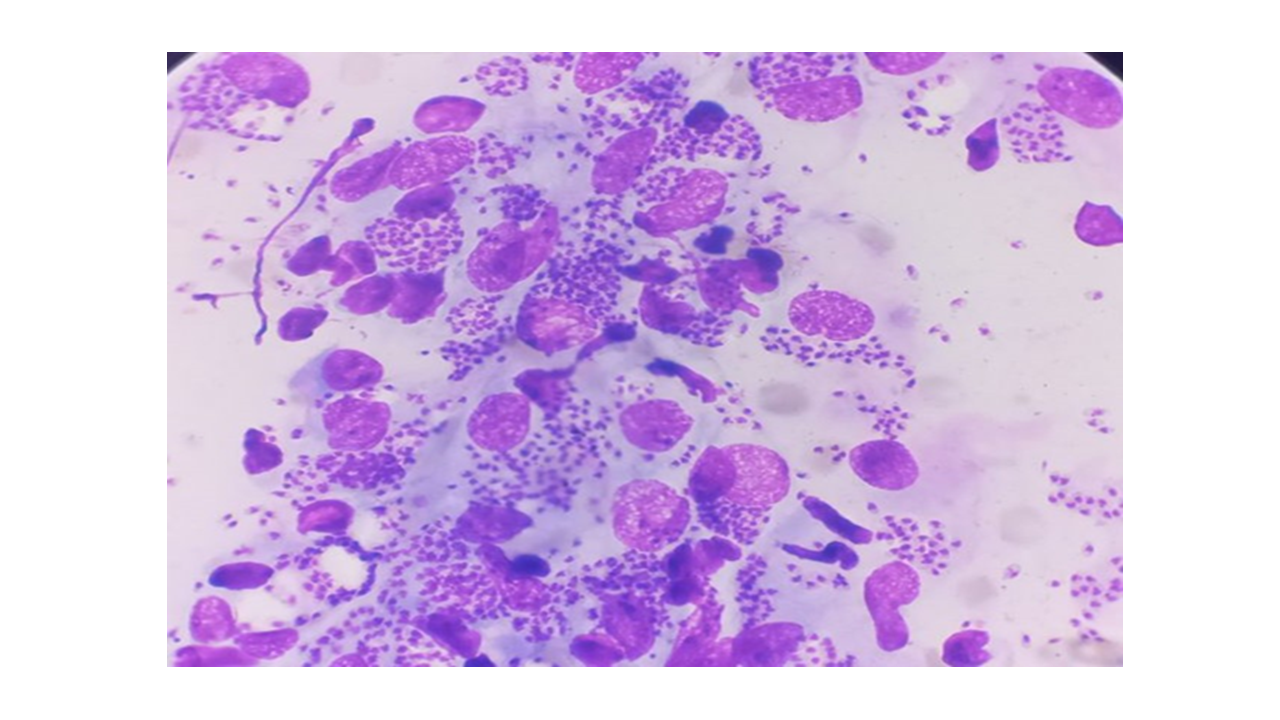

Supplement: S1 Fig — (TIF) [file pntd.0013246.s001.tif]

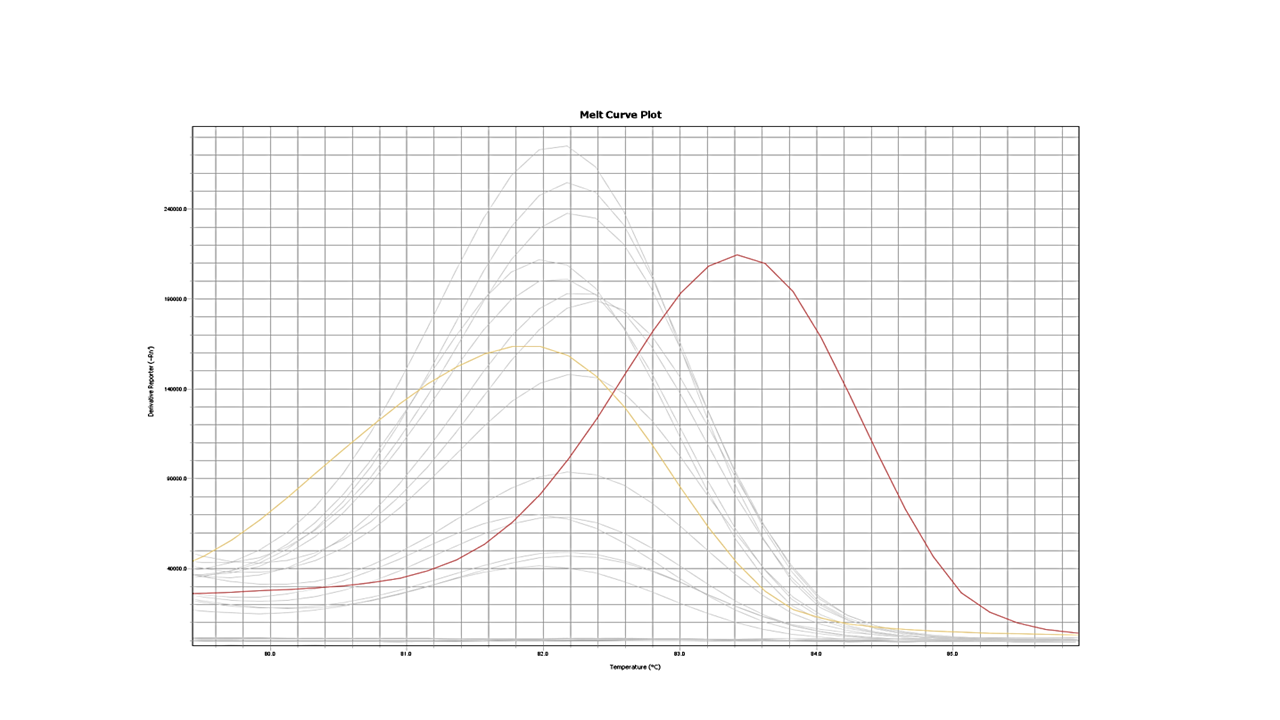

Supplement: S2 Fig — In red is the L. aethiopica positive control, in yellow L. donovani and in grey the samples’ melting curves. Melting temperature (Tm) for L. donovani is 81.8 and for L. aethiopica 83.4. Samples are all situated somewhere in between the Tm values of the two positive controls, where normally L. tropica lies with a Tm of around 82.4, but which was not available for the analysis done in Ethiopia. (TIF) [file pntd.0013246.s002.tif]
