# Supplementary material for: Outbreak of cutaneous leishmaniasis amongst militia members in a non-endemic district under conflict in the lowlands of Somali Region caused by Leishmania tropica, Eastern Ethiopia
Source: PLoS Negl Trop Dis. 2025 Jul 22;19(7):e0013246. doi: 10.1371/journal.pntd.0013246 (PMC12324667; doi:10.1371/journal.pntd.0013246)
Supplement: S1 Table — Fourteen out of 18 positive Leishmania samples provided a melting temperature (Tm). The Tm of samples did not match with that of the positive controls. (DOCX) [file pntd.0013246.s003.docx]

***S1 Table: Raw data of high-resolution melt curves of ITS-1.*** *Fourteen out of 18 positive Leishmania samples provided a melting temperature (Tm). The Tm of samples did not match with that of the positive controls.*

| ***Sample name*** | ***Melting temperature*** |
| --- | --- |
| *S01* | *82.2* |
| *S02* | *82.4* |
| *S04* | *82.3* |
| *S05* | *82.1* |
| *S06* | *82.2* |
| *S07* | *82.3* |
| *S08* | *82.0* |
| *S09* | *82.2* |
| *S11* | *82.0* |
| *S12* | *82.1* |
| *S13* | *82.0* |
| *SH01* | *82.2* |
| *SH02* | *82.1* |
| *SH03* | *82.2* |
| ***L. donovani*** | ***81.8*** |
| ***L. aethiopica*** | ***83.4*** |
