# Supplementary material for: Outbreak of cutaneous leishmaniasis amongst militia members in a non-endemic district under conflict in the lowlands of Somali Region caused by Leishmania tropica, Eastern Ethiopia
Source: PLoS Negl Trop Dis. 2025 Jul 22;19(7):e0013246. doi: 10.1371/journal.pntd.0013246 (PMC12324667; doi:10.1371/journal.pntd.0013246)
Supplement: Attachment — Submitted filename: Comments 24_01571.docx [file pntd.0013246.s004.docx]

**Journal**: PLOS Neglected Tropical Diseases

**Manuscript Number**: PNTD-D-24-01571

**Title of manuscript**: Outbreak of *Leishmania tropica* amongst militia members in a non-endemic district under conflict in the lowlands of Somali Region in Ethiopia

**Article Type: Research Article**

**Comments:**

This research addresses the outbreak of cutaneous leishmaniasis, neglected tropical disease, in non-endemic low altitude setting. It is showed that cutaneous leishmaniasis is getting the problem of low altitude areas. And identified Leishmania tropica as a causative parasite, which is not a common parasite in Ethiopia in previous works. Therefore, it is worth publishing the manuscript for wider circulation and disease control with minor comments presented as follow:

**General comment**:

1. Give page numbers
2. Check the space before and after parenthesis through the whole document.
3. **Title**: It would be better if you can modify the title as “Outbreak of cutaneous leishmaniasis amongst militia members in a non-endemic district under conflict in the lowlands of Somali Region caused by *Leishmania tropica*, Eastern Ethiopia”
4. **Abstract**:
   2.1. Methodology/Principal findings section, line 51. Skin slit and fine needle aspirates…

Better to use skin scrapping instead of skin slit in the whole document. Skin slit better goes with Leprosy

2.2. Methodology/Principal findings section, line 53

Mention that there were a total of 1050 CL patients recruited before the statement “All 1050 patients were male militia members…”

3. Study population of the outbreak investigation section, line 175.
    …providing oral informed consent. Why it was not able to take written informed consent? As it is recommended in research ethics.
4. Outbreak investigation data collection, line 180. Photographs of CL….. it is good to take pictures of CL lesions to characterize and classify patients in different clinical presentations. However it should be included in the patient information sheet and consent form. This has to be also mentioned in the ethical consideration section in the manuscript…
5. Sample collection, Line 185 & 186. Two types of samples were collected: a skin slit at Sitti Primary Hospital and Duunyar Health Center and 186 fine needle aspirates (FNA) at the Sheik Hassen Yabare Comprehensive Specialized Hospital.

Why the two samples were collected from different sites? Do they have different sensitivity, need different expertise or just random?

6. Ethical considerations section, line 219-225.
   6.1. . There shall be written informed consent.
   6.2. The study participants are all adult men. What do you mean by “For individuals aged
   6.3. 223 1 to 17 years….). it seems copied and pasted from elsewhere…
   6.4. As you are dealing with *Leishmania* parasites, you need to have signed the Nagoya protocol from Ethiopian Biodiversity Institute.
   6.5. How did you manage the positive cases? Did they get treated?
7. Result section, line 232 & 233. The statement “The attack rate of the outbreak could not be calculated because the number of involved combatants is classified information” is not clear. And it seems paradoxes that if you did not calculate the attack rate how you set your title as outbreak….? What is your reference to say outbreak?
8. Result section
   8.1. It would be good if you include the lesion size, duration of illness if you have the data
   8.2. Some of the lesions look DCL lesions, DCL lesions usually have non-ulcerative multiple diffused lesions with high parasite load in the lesion. So did you correlate the parasite load with number of lesions and the clinical manifestations?
9. Discussion
   9.1. It would be interesting if you can discuss the findings with recent studies in the Northwest Ethiopia:

<https://journals.plos.org/plosntds/article?id=10.1371/journal.pntd.0012409>, <https://idpjournal.biomedcentral.com/articles/10.1186/s40249-024-01244-x>...

and climate change

<https://www.google.com/search?q=Climatic+and+ecological+factors+responsible+for+distribution+of+Phlebotomine+Sand+flies+and+occurrence+of+Cutaneous+Leishmaniasis%2C+in+Himachal+Pradesh+India&oq=Climatic+and+ecological+factors+responsible+for+distribution+of++Phlebotomine+Sand+flies+and+occurrence+of+Cutaneous+Leishmaniasis%2C+in++Himachal+Pradesh+India&gs_lcrp=EgZjaHJvbWUyBggAEEUYOTIGCAEQRRg90gEJMzQ2MWowajE1qAIIsAIB&sourceid=chrome&ie=UTF-8>

10. Include data availability
