# Supplementary material for: Outbreak of cutaneous leishmaniasis amongst militia members in a non-endemic district under conflict in the lowlands of Somali Region caused by Leishmania tropica, Eastern Ethiopia
Source: PLoS Negl Trop Dis. 2025 Jul 22;19(7):e0013246. doi: 10.1371/journal.pntd.0013246 (PMC12324667; doi:10.1371/journal.pntd.0013246)
Supplement: Attachment — Submitted filename: Response to Reviwers.docx [file pntd.0013246.s006.docx]

Date: 19-March-2025

**To**: PLOS Neglected Tropical Diseases Journal

Manuscript Number: PNTD-D-24-01571

**Subject**: sending Revised manuscript “Outbreak of cutaneous leishmaniasis amongst militia members in a non-endemic district under conflict in the lowlands of Somali Region caused by *Leishmania tropica*, Eastern Ethiopia**”**

Dear Editorial manager,

Thank you for your time reviewing our manuscript. We responded to all comments given by the reviewers and editorial board one by one in track change. To the best of our knowledge, all of the reviewers and editorial board's inquiries have been addressed.

Therefore, we are sending the revised version for consideration. Any correspondence can reach out via [adugnabe@yahoo.com](mailto:adugnabe@yahoo.com).

Sincerely yours,

Adugna Abera, corresponding author.

Dear Reviewers,

We sincerely appreciate the insightful feedback provided on our manuscript. Below, we outline our responses to each comment and the corresponding revisions made in the manuscript.

**Title of manuscript**: Outbreak of cutaneous leishmaniasis amongst militia members in a non-endemic district under conflict in the lowlands of Somali Region caused by *Leishmania tropica*, Eastern Ethiopia

**Article Type: Research Article**

**Comments:**

This research addresses the outbreak of cutaneous leishmaniasis, neglected tropical disease, in non-endemic low altitude setting. It is showed that cutaneous leishmaniasis is getting the problem of low altitude areas. And identified Leishmania tropica as a causative parasite, which is not a common parasite in Ethiopia in previous works. Therefore, it is worth publishing the manuscript for wider circulation and disease control with minor comments presented as follow:

**General comment**:

1. Give page numbers

Thank you, we have added page numbers to the manuscript for ease of reference.

2. Check the space before and after parenthesis through the whole document.

Thank you. All the spaces and parenthesis inconsistence are corrected throughout the manuscript.

3. **Title**: It would be better if you can modify the title as “Outbreak of cutaneous leishmaniasis amongst militia members in a non-endemic district under conflict in the lowlands of Somali Region caused by *Leishmania tropica*, Eastern Ethiopia”

Thank you for your comment. The title has been modified as per the reviewer recommendation.

4. **Abstract**:
   2.1. Methodology/Principal findings section, line 51. Skin slit and fine needle aspirates…

Better to use skin scrapping instead of skin slit in the whole document. Skin slit better goes with Leprosy

The word skin slit changed to ‘skin scraping’ throughout the document.

2.2. Methodology/Principal findings section, line 53

Mention that there were a total of 1050 CL patients recruited before the statement “All 1050 patients were male militia members…”

Thank you for the comment the information suggested is mentioned before the stated statement, see abstract section line 53. Study population of the outbreak investigation section, line 175.
 …providing oral informed consent. Why it was not able to take written informed consent? As it is recommended in research ethics.

Since this study was conducted as part of an outbreak investigation and emergency response, obtaining written informed consent was not necessary and also not feasible. Instead, in accordance with the clinical practice for emergency outbreak intervention oral consent was asked for each patient. Participants with photo taken written informed consent is signed. Finally, we have the IRB approval to use the data.

5. Outbreak investigation data collection, line 180. Photographs of CL….. it is good to take pictures of CL lesions to characterize and classify patients in different clinical presentations. However, it should be included in the patient information sheet and consent form. This has to be also mentioned in the ethical consideration section in the manuscript…

Sample collection, Line 185 & 186. Two types of samples were collected: a skin slit at Sitti Primary Hospital and Duunyar Health Center and 186 fine needle aspirates (FNA) at the Sheik Hassen Yabare Comprehensive Specialized Hospital.

Why the two samples were collected from different sites? Do they have different sensitivity, need different expertise or just random?

Both skin scrapings and fine needle aspirates are preferred methods for collecting samples from the CL lesion. The choice of sample collection method depends on the preference of the health professionals involved. At both hospitals, dermatologists prefer to use fine needle aspirates, while during outbreak investigations and responses, the laboratory technologists typically opt for skin scrapings. Both methods are acceptable for diagnosing CL.

6. Ethical considerations section, line 219-225.
   2.3. . There shall be written informed consent.

As stated earlier, written informed consent was not feasible in an outbreak investigation setting, and we have clarified this in the manuscript.

2.4. The study participants are all adult men. What do you mean by “For individuals aged
2.5. 223 1 to 17 years….). it seems copied and pasted from elsewhere…

The erroneous reference to "1 to 17 years" has been removed, as all participants were adult men (see line 248 & 249).

2.6. As you are dealing with *Leishmania* parasites, you need to have signed the Nagoya protocol from Ethiopian Biodiversity Institute.

The Ethiopian Public Health Institute is responsible and mandated for managing emergency situations, such as outbreaks. EPHI is exempted from signing the Nagoya protocol when the sample sources are outbreak areas. The signed MTA was part of the investigation and response to identify the species and characteristics of the parasite at the genome level for the outbreak.

How did you manage the positive cases? Did they get treated?

Thank you for this comment. The statement about how the study participants get treatment is included in the manuscript, see line 251 & 252.

7. Result section, line 232 & 233. The statement “The attack rate of the outbreak could not be calculated because the number of involved combatants is classified information” is not clear. And it seems paradoxes that if you did not calculate the attack rate how you set your title as outbreak….? What is your reference to say outbreak?

The number of militias deployed to the area was kept confidential, as the information pertains to regional security. Hence, attack rate wasn’t computed. In addition, given the fact that no cases were reported from the areas previously and the sudden occurrence of cases in such a magnitude was taken as an outbreak in accordance with WHO's guidance on defining a CL outbreak. <https://www.emro.who.int/neglected-tropical-diseases/information-resources-leishmaniasis/cl-factsheet.html>.

8. Result section
   8.2. It would be good if you include the lesion size, duration of illness if you have the data
   8.3. Since this is like a retrospective report and plus, most individuals (77%) experienced more than 10 lesions, a detailed clinical record on lesion size is not available. We did not collect the duration of illness for each participant; instead, we recorded the reported day of onset of symptoms and the number of infected individuals using the line list reporting format, see line 255 through 273. Some of the lesions look DCL lesions, DCL lesions usually have non-ulcerative multiple diffused lesions with high parasite load in the lesion. So, did you correlate the parasite load with number of lesions and the clinical manifestations?

The lesion type was classified by an experienced dermatologist but most importantly so far there is no clear criteria for CL clinical classification and as a result we acknowledge the classification is subjective. To the best of our knowledge, we did not have any cases of DCL record.

We have noted a high parasite load, and as mentioned in the results section, there is an atypical presentation of the lesions. We suspect two possibilities: 1) The patient may have experienced multiple bites, and 2) The atypical nature of the lesions could suggest that the parasite is encountering the host for the first time, possibly involving animal reservoirs.

9. Discussion
   9.2. It would be interesting if you can discuss the findings with recent studies in the Northwest Ethiopia:

<https://journals.plos.org/plosntds/article?id=10.1371/journal.pntd.0012409>, <https://idpjournal.biomedcentral.com/articles/10.1186/s40249-024-01244-x>...

and climate change

<https://www.google.com/search?q=Climatic+and+ecological+factors+responsible+for+distribution+of+Phlebotomine+Sand+flies+and+occurrence+of+Cutaneous+Leishmaniasis%2C+in+Himachal+Pradesh+India&oq=Climatic+and+ecological+factors+responsible+for+distribution+of++Phlebotomine+Sand+flies+and+occurrence+of+Cutaneous+Leishmaniasis%2C+in++Himachal+Pradesh+India&gs_lcrp=EgZjaHJvbWUyBggAEEUYOTIGCAEQRRg90gEJMzQ2MWowajE1qAIIsAIB&sourceid=chrome&ie=UTF-8>

I attempted to read the discussion section to incorporate the suggested publication, but I couldn't find space for them.

10. Include data availability

We will include the line list (the data base) as supporting file

Journal Requirements:

1) Thank you for including an Ethics Statement for your study. Please include:

i) A statement that formal consent was obtained (must state whether verbal/written) OR the reason consent was not obtained (e.g. anonymity). NOTE: If child participants, the statement must declare that formal consent was obtained from the parent/guardian.].

Thank you for your comments. The information suggested by the editors are included (Line 250-254).

2) Please upload all main figures as separate Figure files in .tif or .eps format.

All the figures are changed to .tif and uploaded separately.

3) Tables should not be uploaded as individual files. Please remove these files and include the Tables in your manuscript file as editable, cell-based objects.

Thank you, comment accepted.

4) As Fig 5B and 5C have clear photos of individuals, we request you alter the images to ensure that their identities are protected. Please place a black square over the eyes of both persons. Though their eyes are closed, they can still be identified, so adding a box will hopefully prevent this.

Thank you for your feedback. We modified the images by placing a black square over their eyes.

5) Some material included in your submission may be copyrighted. According to PLOSu2019s copyright policy, authors who use figures or other material (e.g., graphics, clipart, maps) from another author or copyright holder must demonstrate or obtain permission to publish this material under the Creative Commons Attribution 4.0 International (CC BY 4.0) License used by PLOS journals. Please closely review the details of PLOSu2019s copyright requirements here: PLOS Licenses and Copyright. If you need to request permissions from a copyright holder, you may use PLOS's Copyright Content Permission form.

Please respond directly to this email and provide any known details concerning your material's license terms and permissions required for reuse, even if you have not yet obtained copyright permissions or are unsure of your material's copyright compatibility. Once you have responded and addressed all other outstanding technical requirements, you may resubmit your manuscript within Editorial Manager.

Potential Copyright Issues:

i) Please confirm (a) that you are the photographer of 2, 4, and 5, or (b) provide written permission from the photographer to publish the photo(s) under our CC BY 4.0 license.

Thank you for your comments. Hailemariam Difabachew is the photographer for images 2, 4, and he is part of the team and co-author of this paper, and he provided oral consent to use the pictures for publication.

ii) Figure 1. Please (a) provide a direct link to the base layer of the map (i.e., the country or region border shape) and ensure this is also included in the figure legend; and (b) provide a link to the terms of use / license information for the base layer image or shapefile. We cannot publish proprietary or copyrighted maps (e.g. Google Maps, Mapquest) and the terms of use for your map base layer must be compatible with our CC BY 4.0 license.

Note: if you created the map in a software program like R or ArcGIS, please locate and indicate the source of the basemap shapefile onto which data has been plotted.

If your map was obtained from a copyrighted source please amend the figure so that the base map used is from an openly available source. Alternatively, please provide explicit written permission from the copyright holder granting you the right to publish the material under our CC BY 4.0 license.
If you are unsure whether you can use a map or not, please do reach out and we will be able to help you. The following websites are good examples of where you can source open access or public domain maps:

* U.S. Geological Survey (USGS) - All maps are in the public domain. (http://www.usgs.gov)

* PlaniGlobe - All maps are published under a Creative Commons license so please cite u201cPlaniGlobe, http://www.planiglobe.com, CC BY 2.0u201d in the image credit after the caption. (http://www.planiglobe.com/?lang=enl)

* Natural Earth - All maps are public domain. (<http://www.naturalearthdata.com/about/terms-of-use/>).

Thank you. The comment for (a) is addressed accordingly see figure 1.

For (b), the map is created using ArcGIS software and source of the basemap shapefile is obtained from open access from UN OCHA, Ethiopia shapefile. For more information, please see the link below. <https://ethiopia.africageoportal.com/maps/5fbc3698295e4a97a015219849ed35ad/about>.

Finally, we deeply appreciate the reviewers’ constructive feedback, which has significantly improved the clarity, scientific rigor, and ethical transparency of our manuscript. All suggested revisions have been incorporated, and we believe the manuscript is now strengthened for publication.

We look forward to your final feedback.
